# *Populus tremula* (European aspen) shows no evidence of sexual dimorphism

**DOI:** 10.1186/s12870-014-0276-5

**Published:** 2014-10-16

**Authors:** Kathryn M Robinson, Nicolas Delhomme, Niklas Mähler, Bastian Schiffthaler, Jenny Önskog, Benedicte R Albrectsen, Pär K Ingvarsson, Torgeir R Hvidsten, Stefan Jansson, Nathaniel R Street

**Affiliations:** Department of Plant Physiology, Umeå Plant Science Centre, Umeå University, 901 87 Umeå, Sweden; Department of Chemistry, Biotechnology and Food Science, Norwegian University of Life Sciences, 1432 Ås, Norway; Department of Plant and Environmental Sciences, University of Copenhagen, Thorvaldsensvej 40, DK 1871 Frederiksberg C, Denmark; Department of Ecology and Environmental Science, Umeå Plant Science Centre, Umeå University, 901 87 Umeå, Sweden

**Keywords:** Sexual dimorphism, RNA-Sequencing, transcriptomics, *Populus tremula*, dioecious

## Abstract

**Background:**

Evolutionary theory suggests that males and females may evolve sexually dimorphic phenotypic and biochemical traits concordant with each sex having different optimal strategies of resource investment to maximise reproductive success and fitness. Such sexual dimorphism would result in sex biased gene expression patterns in non-floral organs for autosomal genes associated with the control and development of such phenotypic traits.

**Results:**

We examined morphological, biochemical and herbivory traits to test for sexually dimorphic resource allocation strategies within collections of sexually mature and immature *Populus tremula* (European aspen) trees. In addition we profiled gene expression in mature leaves of sexually mature wild trees using whole-genome oligonucleotide microarrays and RNA-Sequencing.

**Conclusions:**

We found no evidence of sexual dimorphism or differential resource investment strategies between males and females in either sexually immature or mature trees. Similarly, single-gene differential expression and machine learning approaches revealed no evidence of large-scale sex biased gene expression. However, two significantly differentially expressed genes were identified from the RNA-Seq data, one of which is a robust diagnostic marker of sex in *P. tremula*.

**Electronic supplementary material:**

The online version of this article (doi:10.1186/s12870-014-0276-5) contains supplementary material, which is available to authorized users.

## Background

Sexual dimorphism, the differentiation of both primary (*i.e.* gonads) and secondary (other morphological, behavioural and physiological) sex characteristics is the norm in animal systems [1]. In angiosperms the majority of extant species are co-sexual, being either monoecious or hermaphroditic (*i.e.* they bear separate male and female flowers or have either flowers containing both sexual organs, respectively). However, ~4% of plant species are dioecious [2,3], with different individuals producing only male or female flowers, and it is thought that dioecy evolved from ancestral hermaphrodites, which inherently lack sex chromosomes [4]. In several animal systems including nematodes, insects and mammals, sex determination is well characterised [5], whereas the molecular mechanisms underlying dioecious sex determination in plants remain largely unresolved [4,6]. The emergence of dioecy appears to have occurred relatively recently in many plant species, with sex determining loci being located in small regions of reduced recombination where there may not yet have been adequate time for heteromorphic sex chromosomes to have evolved [4].

Evolutionary theory suggests that sexual dimorphism arises after release from a co-sexual state as each sex adapts to a new fitness optimum following the removal of constraints previously imparted by the other sex – *i.e.* that trade-offs necessarily exist between the male and female functions in a monoecious state [4,7,8]. With the exception of sex-determining loci (or chromosomes), males and females share the same genome. Thus sexually dimorphic phenotypes that are not controlled by genes within the sex determining loci/chromosome must result from differential expression regulation of autosomal genes involved in the development and control of those traits [1]. Examples of expected sexual trade-offs include differential optimal strategies of resource allocation to growth and secondary metabolites (such as phenolic compounds) given production of either pollen or seeds; for example, females may allocate more carbon to secondary metabolites at the expense of stem growth in order to protect seeds from predators and pathogens [9,10], resulting in males and females experiencing contrasting selective pressures [8,11].

The genus *Populus* includes poplars, aspens, and cottonwoods and is a well-established model system [12] with a high quality genome sequence available for *P. trichocarpa* [13,14]. *Populus* species and hybrids have numerous industrial and silvicultural uses [15,16] and are often keystone species [17,18]. In *Populus,* dioecy is the common condition with the only exception being the monoecious, hermaphroditic *P. lasiocarpa* (see citations in [19]). There are also rare cases of gender reversion, perfect (bisexual) flower formation and even mature seed catkin formation on male trees [19-21 and citations in 22]. *Populus* species do not have heteromorphic sex specific chromosomes [22], and the molecular mechanism of sex determination remains undetermined, although sex is genetically determined [23]. In *P. trichocarpa* there is substantial evidence that the sex-determining locus is located in the peritelomeric region of chromosome 19 [22,23]. For all *Populus* genetic maps where sex has been included as a marker during map construction, there is always a single sex-linked locus that is located on chromosome 19. However, its location on that chromosome varies in different sections of the genus. There are also contrasting reports as to which sex is heterogametic [22,24-26]. In the aspens it is now well established that the sex determination locus is located in the pericentromeric region of chromosome 19 [24-28]. Pakull *et al*. [28] recently identified that Potri.019G047300, a gene that the same group had previously identified as a candidate in the sex determination locus [24], is either completely or partially deleted specifically in females, a finding that we independently discovered and detail below.

There is a current lack of knowledge of whether global or specific patterns of sex biased gene expression exist in non-reproductive tissues of dioecious plant species [4]. To date, this has been investigated in a single study of *Silene latifolia* [29], which considered only 22 ESTs. Here we addressed this question using *P. tremula,* which produces high amounts of phenolic-based secondary metabolites that have been implicated in defence against herbivores and pathogens [30,31] making it a suitable model system to test for sexually dimorphic differences in resource allocation to growth and defence. We explored global gene expression patterns in combination with a set of diagnostic phenotypes in non-reproductive tissues (leaves) of sexually mature *P. tremula*. The same phenotypes were additionally assayed in sexually immature trees. Gene expression was profiled using both whole genome oligonucleotide microarrays and RNA-Sequencing (RNA-Seq). The expression data were used for both individual gene differential expression tests as well as a machine learning approach to test for genomic regions containing combinations of genes exhibiting sex-related expression differences.

## Results

### *Phenotypic analysis reveals no evidence of sexual dimorphism in* P. tremula

**Figure 1:**
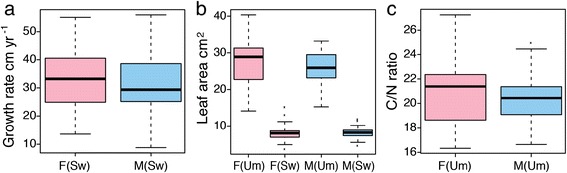
**Growth and resource allocation in male and female** ***Populus temula*** **trees.** Boxes representing females (F) are coloured pink and males (M) coloured blue, in the Umeå Aspen (Um) and Swedish Aspen (Sw) collections. **(a)** Growth rate calculated as height increment over five years in SwAsp. Analysis of Variance (ANOVA) results showed no significant sex differences (F_1,45_ = 0.448, P =0.507) **(b)** Individual leaf area in UmAsp and SwAsp. ANOVA results showed no significant sex differences for samples from either the Um (F_1,38_ = 0.958, P =0.334) or Sw (F_1,44_ = 0.012, P =0.914) collections. **(c)** Foliar carbon/nitrogen ratio in Um. ANOVA results showed no significant sex differences (F_1,38_ = 0.631, P =0.432).

**Figure 2:**
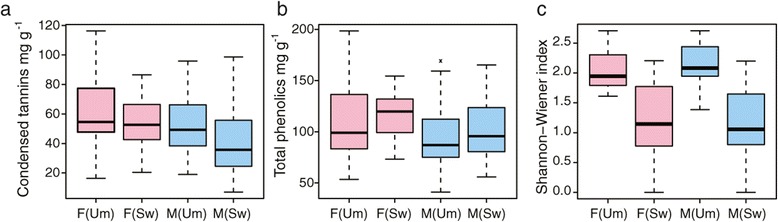
**Secondary metabolite and herbivory phenotypes in male and female** ***Populus tremula*** **in the Umeå Aspen (Um) and Swedish Aspen (Sw) collections.** Boxes representing females (F) are coloured pink and males (M) coloured blue. **(a)** Foliar condensed tannins. Analysis of Variance (ANOVA) results showed no significant sex differences for samples from either the Um (F_1,38_ = 1.667, P =0.203) or Sw (F_1,45_ = 2.764, P =0.103) collections **(b)** Foliar total phenolic concentrations. ANOVA results showed no significant sex differences for samples from either the Um (F_1,38_ = 01941, P =0.172) or Sw (F_1,45_ = 2.561, P =0.117) collections. **(c)** Shannon-Wiener index of arthropod herbivore diversity. ANOVA results showed no significant sex differences for samples from either the Um (F_1,38_ = 0.659 P =0.422) or Sw (F_1,45_ = 0.074, P =0.787) collections.

We found no evidence of sexual dimorphism in tree height or diameter (Additional file 1) in either the Umeå Aspen collection (UmAsp; [32]) or the Swedish Aspen (SwAsp; [33]) samples or for height increment, a measure of vigour, in the juvenile SwAsp samples (Figure 1a, Additional file 1). Similarly, we found no statistical evidence of sexual dimorphism for leaf area (Figure 1b), leaf nutritional quality (nitrogen and carbon content and their ratio, Figure 1c) or specific secondary metabolites (total phenolics and condensed tannins, Figure 2a-b) in either the UmAsp or SwAsp samples (Additional file 1). All SwAsp phenotypic data except carbon and nitrogen concentration were generated by Robinson *et al*. [34], who showed that these, and other, traits had a substantial degree of heritability (clonal repeatability), a result that could only be obtained from high quality phenotypic data, negating the possibility that the observed lack of significant sexual dimorphism resulted from low data quality.

### Herbivorous insects display no sexual preference

Arthropods are common folivores on *P. tremula* and numerous aspen-associated morphospecies have been recorded [34]. We found no statistically significant sex-related differences for arthropod abundance, species richness, feeding guild abundances, or the Shannon-Wiener diversity index in either the UmAsp or SwAsp samples (Figure 2c, Additional file 1). We also found no statistically significant sex-related differences in the arthropod community of UmAsp and SwAsp analysed by non-parametric Multivarite Analysis of Variance (MANOVA; UmAsp: F_1,38_ = 0.325, P =0.808; SwAsp: F_1,45_ = 0.825, P =0.5, Additional file 1).

### Transcript profiling reveals no global patterns of sex-biased expression

**Figure 3:**
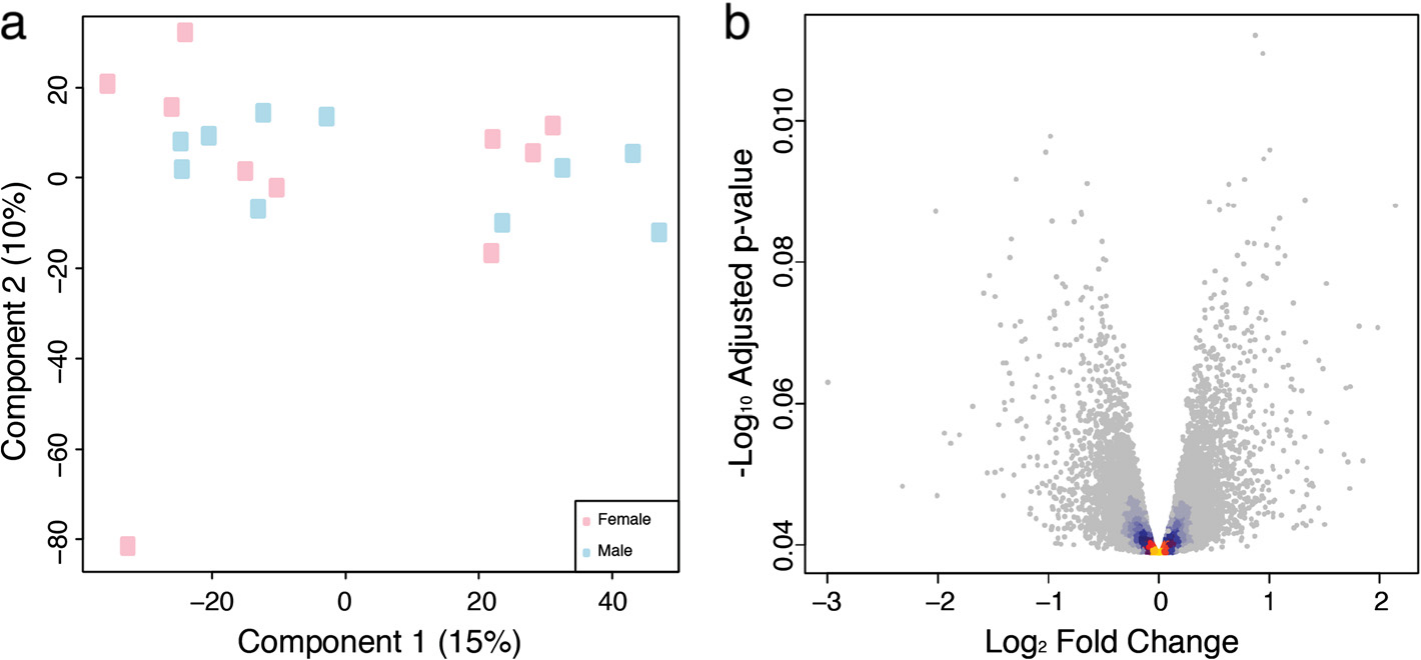
**Overview of microarray gene expression patterns in male and female** ***Populus tremula*** **trees from the Umeå Aspen collection. (a)** Principal Component Analysis plot of the microarray data with samples classified by sex (male in blue, female in pink). The percentage variance explained by each component is shown in parenthesis for each axis. The female sample shown at the bottom left of the plot was classified as an outlier and excluded from statistical analyses. **(b)** Volcano plot of the negative log_10_ p-value (y-axis) plotted against log_2_-fold change (x-axis) showing the results of differential expression analysis comparing male to female trees assayed using whole-genome Agilent oligonucleotide microarrays. Technical noise was accounted for in the statistical model by including factors for slide and sub-array within slide and the effect of sex was tested after removal of variance due to those technical effects. Non-significant genes are coloured to indicate density, which is shaded from yellow (high) to blue (low). No genes were significant (note that 0.01 on the y-axis corresponds to a p-value of 0.977).

**Figure 4:**
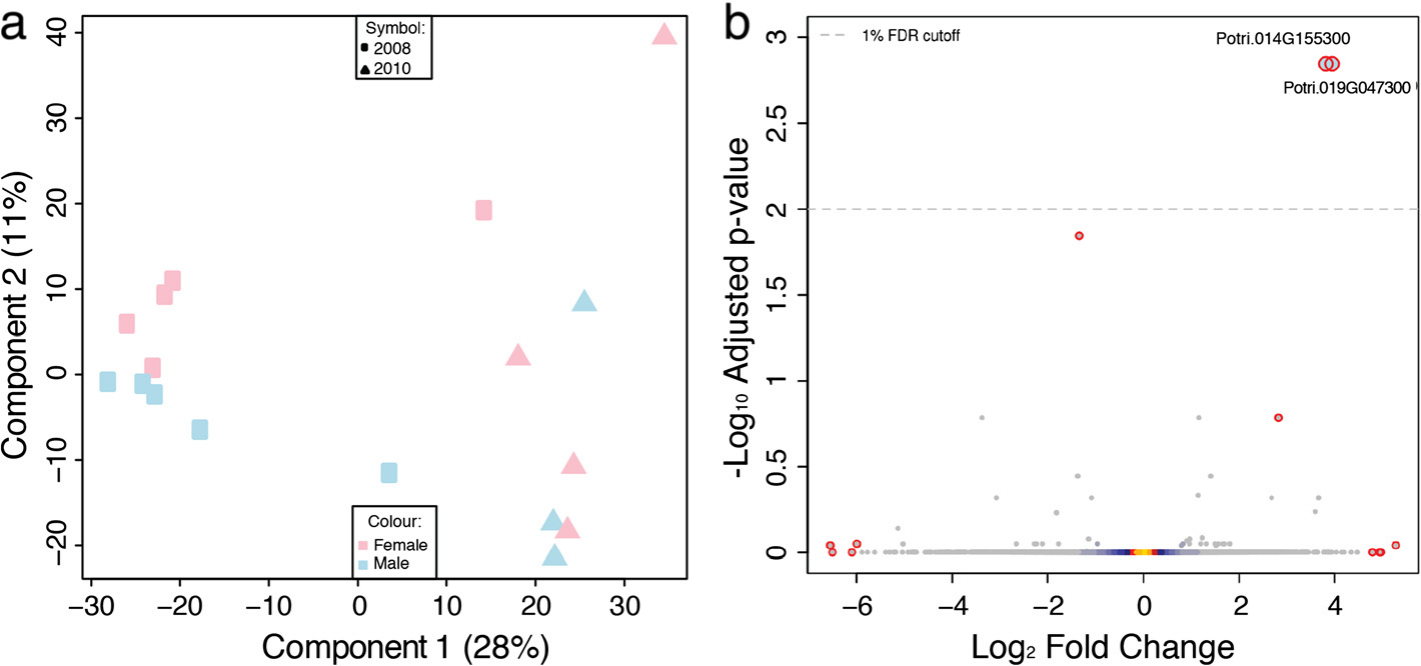
**Overview of RNA-Sequencing gene expression patterns in male and female** ***Populus tremula*** **trees from the Umeå Aspen collection. (a)** Principal Component Analysis plot of the RNA-Sequencing (RNA-Seq) expression data with samples classified by sex (male in blue, female in pink) and by year of sampling (2008 as squares and 2010 as triangles). The percentage variance explained by each component is shown in parenthesis for each axis. **(b)** Volcano plot of the negative log_10_ p-value (y-axis) (*i.e.* the log odds ratio) plotted against log_2_-fold change (x-axis) showing the results of differential expression analysis assayed using RNA-Seq comparing male to female trees. The statistical model included factors for year of sampling and sex and the effect of sex was tested after removal of the year effect. Significant genes are shown in blue where expression was higher in males. For the two significant genes at a 1% False Discover Rate (FDR) cut-off, the obtained p-value was <1e^-10^ and was therefore set to 0. As a result the log odds value is infinite and was therefore replaced with the next largest log odds +1. Non-significant genes are coloured to indicate density, which is shaded from yellow (high) to blue (low). The dashed horizontal line represents a 1% FDR. The four genes with the smallest p-values (regardless of significance) and the four genes with the highest and lowest non-significant fold change values are circled in red. These genes are represented in Additional file 2. The gene identifiers for the two statistically significant genes are shown (identifiers refer to V3 of the *P. trichocarpa* genome).

**Figure 5:**
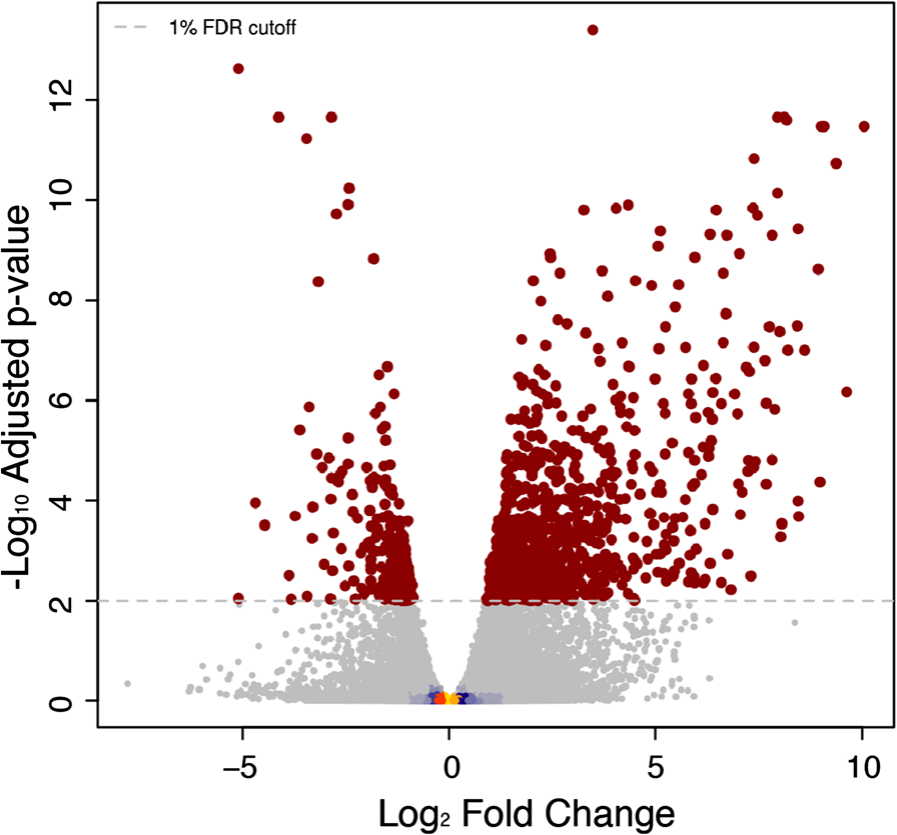
**Differential expression of year of sampling effect.** Volcano plot of the negative log_10_ p-value (y-axis) - *i.e.* log odds ratio - plotted against log_2_-fold change (x-axis) showing the results of differential expression analysis assayed using RNA-Sequencing comparing samples collected from trees in 2008 and 2010. Gene expression was assayed using samples collected from trees in 2008 and 2010 by RNA-Sequencing. Significant genes are shown as larger brown points. Non-significant genes are coloured to indicate density, which is shaded from yellow (high) to blue (low). The dashed horizontal line represents a 1% False Discovery Rate.

We profiled gene expression in mature leaves of male and female *P. tremula* from the UmAsp collection using whole genome oligonucleotide microarrays (Figure 3) and RNA-Sequencing (RNA-Seq; (Figure 4). The samples used for RNA-Seq profiling were collected in two years and a Principle Component Analysis (PCA) analysis revealed clear differences between samples from the two years (Figure 4a). A total of 1,138 genes were identified as significantly differentially expressed between years (Figure 5).

Despite many genes having relatively high mean fold-changes between sexes in the RNA-Seq data (Figure 4b), the within-sex variation for those genes was high resulting in non-significant statistical test results. To further explore this, we examined the variance among samples for the four genes with the lowest and highest fold change values and for the four genes with the smallest p values regardless of fold change (of which only two were statistically significant) in the RNA-Seq data. Variance for genes with high between-sex fold-change values was high (Additional file 2) and only two genes (see below) were statistically significantly differentially expressed between males and females.

We applied a machine learning approach, support vector machines (SVMs), to sliding windows of contiguous genes in the *P trichocarpa* genome to identify any regions where the combination of expression patterns for all genes within the window were predictive of sex. No statistically significant gene combinations that were predictive of sex were identified.

### Potri.019G047300 is not present in females and is located in the sex determination locus

In contrast to the clear influence resulting from year of sampling, differential expression analysis identified only two statistically significant sexually dimorphic differences in the RNA-Seq dataset (Figure 4b; Potri.014G155300, FDR adjusted p-value 0.00: Potri.019G047300, FDR adjusted p-value 0.00) and none in the microarray dataset (Figure 3b). These two genes were not represented in the v1.1 genome annotation that was used for the array design, therefore excluding the possibility to cross-validate the result in the microarray dataset. However, Pakull *et al*. [28] provide an excellent and completely independent confirmation of this finding for Potri.019G047300.

**Figure 6:**
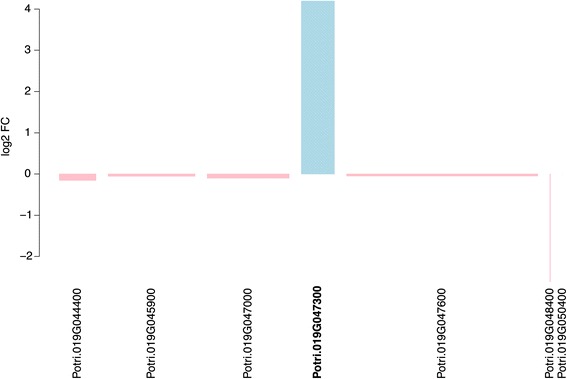
**Bar graph showing differential expression of seven candidate genes from the sex determination locus of** ***Populus tremuloides*** **[** 24 **].** The width of each bar indicates the mean expression level of each gene relative to the gene with the highest expression (Potri.019G047600), which had an expression value of 240, such that narrow bars represent low expression and wide bars high expression. The y-axis indicates the log_2_ fold-change between male and female trees. Expression values represent variance stabilising transformation normalised read counts. Genes displaying higher expression in females are shown in pink and those with higher expression in males in blue. The gene model identifier for the only one of these genes that was identified as significantly differentially expressed in the RNA-Seq data is marked in bold text.

Potri.014G155300 has no functional annotation but contains Pfam (Protein family) domains associated with cellulose synthase activity. This gene has highest sequence similarity to the *Arabidopsis thaliana* homolog AT2G32540, which is annotated as “Cellulose synthase-like B4”. More interestingly, the second gene (Potri.019G047300) is one of seven candidate genes identified within the sex determination locus of *P. tremuloides* by Kersten *et al*. [24] and was recently shown by the same authors to have a partial or complete deletion in female aspens [28] resulting in expression only being observed in males. The gene has no current functional description in poplar but contains WD40 domains and shows highest sequence similarity based homology to the *A. thaliana* gene AT5G16750 (TORMOZEMBRYO DEFECTIVE, TOZ). In *A. thaliana* this gene is required for regulated division planes and embryo development [35] and is thought to be involved in 18S rRNA biogenesis and RNA methylation. We examined the expression of the seven candidates highlighted by Kersten *et al*. [24] within our data, revealing that this was the only gene displaying any evidence of differential expression between sexes (Figure 6). The gene was expressed more highly in male than female trees. Examination of Affymetrix gene expression microarray data represented at the poplar eFP resource (http://bar.utoronto.ca/efppop/cgi-bin/efpWeb.cgi; [36]) shows that this gene has high expression in male catkins and low expression in female catkins for the three array probes representing this gene (PtpAffx.113801.1.S1_s_at, PtpAffx.212175.1.S1_at; probe-to-gene links were obtained from PopArray [37], http://aspendb.uga.edu/). However, as these data represent expression in *P. balsamifera* and as this gene is not deleted in female *P. trichocarpa* trees (as suggested by the presence of the complete gene structure in the assembled genome sequence) these results require caution for extrapolation to the aspens.

**Figure 7:**
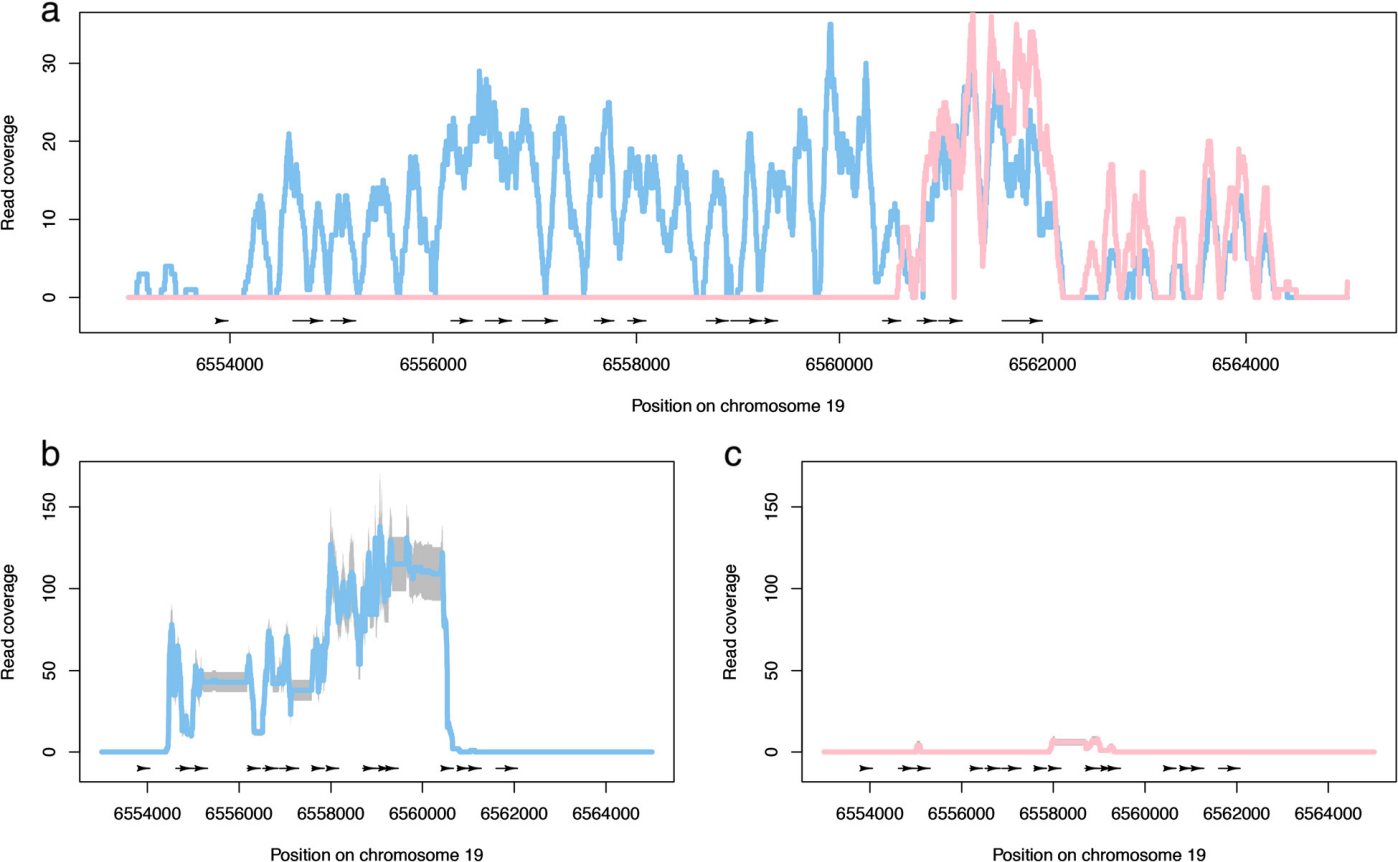
**Genomic DNA and RNA-Sequencing read coverage (y axis) for the region of chromosome 19 (x axis) including Potri.019G047300. (a)** Read coverage of uniquely mapping genomic DNA reads from a male (blue, 229.1) and female (pink, 349.2) individual. Black arrows represent exons with arrow direction indicating strand. **(b,c)** Read coverage of uniquely mapping RNA-Sequencing reads for male (b, n = 8) and female (c, n = 9) individuals. The coloured line represents the average per base pair read coverage across all individuals with grey indicating ± two standard deviations.

We used genomic re-sequencing data (collected for another study, but available on request) from two of the assayed trees, one male and one female, to further explore this locus. Genomic DNA sequencing reads (2x100 bp paired-end reads generated from a 300 bp insert library and sequenced using standard procedures on the Illumina HiSeq 2000 platform) were aligned to the reference *P. trichocarpa* genome sequence and only uniquely mapping reads were considered. This revealed that there is a deletion of this region in the female individual (Figure 7), which is in agreement with the results recently reported by Pakull *et al*. [28] and that explains the lack of any RNA-Seq reads being produced from female individuals in this region. As such females appear to be homozygous for absence of this locus. Corresponding plots based on RNA-Seq reads from all individuals assayed are available in Additional file 3. A single female individual (226.1) showed expression of the TOZ gene. We have been unable to confirm the sex of this tree as it has not flowered again since sex was originally determined. Repeating the above analyses with or without this individual did not affect the results obtained (see the R analysis HTML report on the PopGenIE FTP site [38]).

### *No evidence of biased sex ratio in* P. tremula

We observed no sex bias in the *P. tremula* collections studied. The sex ratio of the SwAsp samples was 1:1 (female:male, where 52 trees of a total 116 in the collection are of known sex, Additional file 4). In the UmAsp samples the sex ratio was 1:1.1 (where 42 trees of 350 are of known sex, Additional file 4).

## Discussion

In dioecious species, evolutionary theory suggests that males and females may have contrasting optimal strategies of resource investment to maximise reproductive success. As a result, natural selection would result in the emergence of sexual dimorphism in phenotypic, biochemical and ecological traits associated with contrasting resource allocation and utilisation as each sex evolves towards fitness optima. If phenotypic sexual dimorphism does arise, there will be concomitant dimorphism in gene expression patterns in the corresponding tissue(s) associated with those phenotypic traits. Such dimorphic gene expression patterns will be independent of any differential gene expression associated with sex determination and the control of reproductive tissue development. As such, although those genes may in some cases be located within the sex determination region or chromosome, it is likely that many such regulated genes will be autosomal.

In the current study our primary interest was to test the hypothesis that male and female *P. tremula* individuals invest resources differentially, resulting in sexual dimorphism. To this end a number of morphological and biochemical traits (Additional file 1) were selected to be diagnostic of such dimorphism in leaves sampled from a set of wild-growing, sexually mature *P. tremula* individuals (the UmAsp collection) and a set of common-garden, sexually immature and clonally replicated individuals (the SwAsp collection, see materials and methods). We focused on leaves as these are the primary point of interaction between aspens and the majority of their associated herbivores as well as representing the site of energy assimilation and therefore carbohydrate production for utilisation in primary (growth-associated) and secondary metabolism.

### P. tremula *shows no phenotypic evidence of sexual dimorphism*

Height and diameter are often used as proxies for fitness based on the assumption that faster growing and larger individuals are better equipped to out-compete their neighbours, allowing greater resource acquisition that can be invested in sexual reproduction [39]. We found no statistical evidence supporting phenotypic differences between males and females for any of the phenotypic traits that we assayed in either the sexually mature UmAsp or sexually immature SwAsp samples. These results contrast with observations in Pauley [40] who reported a strong male biased sex ratio within a collection of superior-growth individuals of five North American *Populus* species. This was interpreted as potential evidence that males may display more vigorous growth. In *P. euphratica* growth traits showed variable differences between sexes among sample plots with no consistent statistically significant difference between sexes for assayed growth traits [41]. In the cross-species meta-analysis presented in Cornelissen & Stillin [10], males in general exhibited larger leaves, lower concentrations of secondary metabolites and higher growth rates. However, and in agreement with our results, there was no sexual dimorphism for height or nutrient concentrations. In *P. deltoides,* Farmer [42] observed that males were taller than females but did not have greater stem diameter. Citations within Farmer detail observations that the height of *P. tremula* x *P. tremuloides* seedling cohorts was correlated to the proportion of males, but also that no differences in vigour between sexes had been identified in *P. tremuloides*. Our results are also in agreement with those reported for *P. tremuloides* by Mitton & Grant [43] and Stevens & Esser [44]. Based on the current limited number of publications examining sexual dimorphism we would conclude that it is not yet possible to ascertain whether any generalisations can be formed regarding the presence or absence of sexual dimorphism for growth or defence related traits in *Populus.*

Several studies have additionally reported higher herbivore loads associated with increased growth in males [45-48], however we found no such reports in *Populus*. Although the meta-analysis presented in Cornelissen & Stiling [10] found that, in general, males suffered higher arthropod abundances, showed evidence of reduced levels of secondary metabolites and increased growth rates, it is not possible to extrapolate such generalised findings as being relevant to a specific species. Our own data identified no statistical evidence of sexual dimorphism in arthropod abundance, diversity or folivore herbivory damage in *P. tremula* in concordance with a lack of dimorphism in assayed growth and defence related phenotypes.

The majority of current evidence for sexual dimorphism in *Populus* has been identified in response to stressful environmental conditions, for example under drought, salinity [49-51], UV-B radiation [52], chilling stress [53], or differential nutrient availability [54-57] where females were found to be more sensitive. However, these studies typically used small sample sizes, in some cases being restricted to only a single individual of either sex. They also profile response to short term, acute stress exposure in most cases. This is in contrast to the approach taken here where we sample a collection of wild-growing trees. In these conditions individuals would have been exposed to various short to long-term stress events. We were interested to know whether evidence of dimorphism is present under such conditions in addition to knowing if there is evidence of sexual dimorphism for resource allocation to growth in sexually immature trees. In *Salix* it has been reported that evidence for sexual dimorphism varies through the growing season [11]. Such reports can lead to the general impression that sexual dimorphism is common or expected. However, bias against the publication of negative results potentially means that many such examples of a lack of dimorphism have remained unreported. The variable presence of evidence for sexual dimorphism also cautions against over-extrapolation of such results until multiple conditions and seasonal sampling points have been considered for each species and each geographic area of interest.

At both the national (SwAsp) and local (UmAsp) scales we believe that our sampling represents an unbiased representation of wild-growing mature trees, with sampling taking place with no knowledge of, or consideration for, sex or the presence of flowering. It is, of course, possible that studies testing more specific hypotheses, for example along an elevational cline (as reported for *Salix* [11]), may uncover evidence for shifting sex ratios or for sexual dimorphism. Indeed we see weak evidence for this within the SwAsp collection (Additional file 4) suggesting that further studies are needed in *P. tremula* before general conclusions can be drawn. We would caution against extrapolation of these findings beyond *P. tremula* growing in natural conditions within the geographic range covered by our sampling. To allow more general conclusions to be drawn for other *Populus* species, members of the Salicaeae and, more widely, other dioecious herbaceous species, will require equivalently detailed investigation and publication.

### Environment affected gene expression more than sex

We profiled gene expression in leaves of sexually mature *P. tremula* individuals from the UmAsp collection to test the hypothesis that sexually dimorphic phenotypic traits would also be revealed by concomitant differential gene expression between males and females in non-reproductive tissues for genes associated with those phenotypes. In agreement with the above morphological and biochemical phenotypic results, we found no reliable evidence of large-scale sexually dimorphic (sex-biased) differential expression (Figures 3 and 4). In contrast, clear evidence of an effect of sampling collection was found (Figures 4a and 5). As samples from the two years were collected on different dates and from different heights within the canopy we cannot determine whether environmental/climatic variation between years or height in the canopy accounted for this difference. Significantly differentially expressed genes between the sample collections were over-represented for Gene Ontology (GO) biological process categories primarily involved in cellulose biosynthesis and glucan and lipid metabolism, most likely reflecting the slightly different sampling dates, with year-to-year variance in climatic conditions affecting the rate of leaf development and maturity. This exemplifies that in *P. tremula* leaves, changes in environmental conditions influence expression to a greater extent than the sex of an individual and that our expression data was of sufficient quality to identify biological effects influencing gene expression patterns.

The primary aim of this study was to identify patterns of sexually dimorphic gene expression associated with the morphological and biochemical traits profiled. As such, we would have expected relatively large numbers of genes to be involved should dimorphism have been present. For example, if females invest more resources into chemical defences produced via secondary metabolism, there would be corresponding sexually dimorphic differences in the expression of genes involved in secondary metabolism. Here we present gene expression results generated using *P. tremula* RNA-Seq read alignments to the *P. trichocarpa* reference genome. On the basis of a number of considered factors we do not believe that this biased our results: firstly, the vast majority - over 90% - of RNA-Seq reads aligned to the *P. trichocarpa* genome, suggesting that the two species have an almost entirely overlapping gene space and that sequence divergence within coding regions is not high enough to impact read alignment; secondly, we have also used a draft assembly of the *P. tremula* genome (available at the PopGenIE FTP resource [38]; ftp://popgenie.org/popgenie/UPSC_genomes/UPSC_Draft_Assemblies/Current/Genome/) to confirm that the vast majority of annotated CDS regions in *P. trichocarpa* can be aligned to the draft assembly and that analysis of the RNA-Seq data aligned to this draft genome does not produce different results; lastly, alignment of *P. tremuloides* and *P. tremula* x *P. tremuloides* genetic maps to the *P. trichocarpa* chromosomes suggests that there have been no major genome rearrangements between aspens and *P. trichocarpa* [24,27], although micro-synteny has not been examined to date. As such, although there may be a small number of genes unique to, or highly variable between, each species, differences between the two species are not sufficient to affect the results of global-scale expression pattern analyses. We would caution that studies aiming specifically to identify the gene(s) underlying sex determination, where genetic mapping suggests a single locus is involved and for which a single or small number of genes are likely involved, could substantially benefit from use of species-specific genome sequences.

### Potri.019G047300 *is absent in females and is located in the sex determination locus*

The proposed peritelomeric sex determination locus on chromosome 19 of *P. trichocarpa* represents a region of reduced recombination [23]. Kersten *et al*. [24] recently provided evidence of a similar region of reduced recombination in the pericentromeric sex-linked locus of chromosome 19 in *P. tremuloides*. One of the two genes that we identified as being highly, and exclusively, significantly differentially expressed between sexes in the RNA-Seq data (Potri.019G047300) is located in that identified sex determination locus of *P. tremuloides*. It is one of seven candidate genes identified by Kersten *et al.* [24] on the basis of Gene Ontology and other annotation evidence as having the potential to be involved in sex determination, primarily due to annotated involvement in floral organ development. This was the only one of those seven genes with evidence of differential expression between sexes in our data (Figure 6). Pakull *et al*. [28] recently refined this finding, reporting a complete or partial deletion of this gene in female *P. tremuloides* and *P. tremula* individuals. Here we present independent confirmation of this finding, supported by both genomic DNA and RNA-Seq results (Figure 7). It is unclear what the biological influence of differential expression of the gene in leaves might be. Our results clearly show that this single gene did not result in any larger-scale downstream patterns of sex-biased expression and examination of expression evidence at the PopGenIE [58] org and poplar eFP resources showed that expression of this gene varies between tissues and through the growth cycle, suggesting that expression is not merely constitutively fixed in males. This is certainly a finding that deserves future attention.

Due to reduced recombination rates in sex-determination loci, all genes within a locus will, on average, be co-inherited [22]. Such a case could be identifiable as a region of the genome where a contiguous set of genes would have consistently sex-biased expression, resulting from either presence/absence differences for genes present only in the W-linked (or Y-linked) haplotype, or expression level differences for genes present in both haplotypes, but with fixed *cis*-acting differences between the Z and W (or X and Y) haplotypes. As the degree of expression bias may be small on a gene-by-gene basis, single gene analysis methods may lack the sensitivity to detect such differences but methods considering combinations of genes may succeed. For example, such a situation could have been possible for all seven of the candidate genes in the *P. tremuloides* sex determination locus discussed above. We therefore applied a machine learning approach to identify any sets of collinear genes (within sliding windows) that were predictive of sex. However, no statistically significant combinations of weakly predictive genes or synergistically predictive genes were identified.

## Conclusions

We present an assessment of sex ratio and the lack of sexual dimorphism based on two independent samplings of Swedish *P. tremula*. Our sample of 87 was more comprehensive than almost all previous such assessments in *Populus* and, as such, we feel that the results obtained are an accurate representation for *P. tremula*. We identified no evidence that sex has served as a significant selective pressure affecting gross-scale morphological, biochemical or herbivorous insect interaction traits expected to be diagnostic of differential resource investment and allocation strategies. Correspondingly, there was no evidence for sex-biased patterns of gene expression associated with those, or any other, traits.

Although no evidence of large-scale patterns of sexually dimorphic gene expression patterns were identified, a previously identified candidate gene for sex determination in *P. tremuloides* [24] showed exclusive expression in males due to the homozygous absence of the locus in female individuals, an observation warranting future attention.

## Methods

### Phenotypic, morphological and biochemical traits

We examined the incidence of flowering in a collection of (sexually mature) wild, mature aspen (*Populus tremula* L.) trees in Sweden, the Umeå Aspen collection (UmAsp; [32]). In addition we used sexually immature clonal copies of trees of known sex from the Swedish Aspen (SwAsp) collection growing in a common garden experiment near Sävar, Umeå in Sweden, that were propagated and planted as described previously [33].

#### Umeå aspen collection (UmAsp)

Twenty-two trees bore male flowers and 20 trees bore female flowers in the spring of 2007. Tree sex was determined by visual examination of catkins and was confirmed by returning to each tree to record whether female trees retained catkins post pollination when male catkins had died. A description of trees and their geographic coordinates, together with sampling dates, is provided in Additional file 5. Tree height was measured in 2007 (when the collection was established) using a vertex dendrometer and trunk circumference was measured at breast height (1.3 m). Sampling took place on 22-25 June 2008. Six branches, each bearing approximately 60 leaves, were cut 4-5 m above ground level, in a transect from east to west across the canopy, or the nearest feasible positions, for morphological and herbivore community analyses. Sampled branches were sealed into plastic bags and kept at 4°C prior to morphological and arthropod analyses. A second sample of ten undamaged leaves from the west of the canopy was frozen in liquid nitrogen and stored at -80°C prior to RNA extraction. Following RNA extraction, samples were freeze-dried and used in assays of total phenolics and condensed tannins as described in [34] and leaf carbon and nitrogen against aspartame, wheat and atropine standards (Flash EA 1112 NC Soil Analyser, Thermo Fisher Scientific, Milan). Ten undamaged leaves were taken from each branch sample and scanned for image analysis conducted with LAMINA [59] to obtain leaf area. The same ten leaves were dried and weighed to calculate specific leaf area. From each sample bag, forty leaves were removed at random and examined for arthropod herbivore specimens and leaf modifications caused by known arthropods on aspen [34], from which arthropod species richness was calculated as the total number of morphospecies and arthropod abundance as the total number of individuals. Herbivores were also classified and summed by feeding guilds based on utilisation of the plant tissue: leaf-chewers, leaf-miners, gall-makers and leaf-rollers. Arthropod herbivore diversity was calculated for each genotype with the Shannon-Wiener index [60] using the diversity function from the package vegan [61] in R [62].

#### Swedish aspen collection (SwAsp)

In the Sävar common garden, clonal copies of 23 genotypes within the SwAsp collection originated from female and 24 from male trees. Sex was determined based on available flowering observations of the original trees from which the common garden trees were cloned. To determine sex, catkins were removed and examined using a binocular microscope. The cloned trees in this common garden experiment have not yet reached sexual maturity. Trees were measured annually in autumn for height with a measuring pole and for diameter at 30 cm from ground level using digital calipers. Growth rate over a five year period was calculated as (Height (2011) – Height (2006))/5, when the trees were between two and seven years old. Ten undamaged, mature leaves were harvested for measurement of leaf area and specific leaf area (leaf area/dry mass), and a further ten leaves were harvested, dried and assayed for condensed tannins and total phenolics as described in [34]. Leaf nitrogen and carbon content were analysed on an available subset of six male and six female genotypes harvested on 29 June 2010. Counts of all arthropod herbivores on each tree in the SwAsp common garden were conducted on 27 – 29 June 2008 as described in [34]. Morphospecies of folivorous arthropods were summed from the replicates of each genotype. Arthropod species richness was calculated as the sum of arthropod morphospecies on each SwAsp genotype. Arthropod herbivore diversity (Shannon-Wiener index), abundance, species richness and feeding guild abundances were calculated on each genotype using the same methods as the UmAsp samples. Details of clone geographic origins, sex, replication in the common garden and phenotypic data collected are provided in Additional file 5.

### Statistical analysis

Statistical analyses were conducted and figures generated in R conducted [R Core Development Team reference]. Statistical significance for all tests was determined at α ≤0.05. Dependent variables (tree phenotypes) were tested for normality and homogeneity of variance using Anderson-Darling and equal variance (Bartlett) tests to meet the assumptions of analysis of variance (ANOVA). Where transformation using Box-Cox powers or log-transformation did not result in improvement of the distribution of a dependent variable, a two-tailed Mann–Whitney *U*-test was applied. In SwAsp, the latitude of origin for each genotype was initially applied as a covariate, to account for phenotypic variation associated with latitude, however no significant effect of sex was identified for any response variable (P >0.1), therefore final analyses were conducted without a covariate. ANOVA or Mann–Whitney U-tests tested the effect of sex (independent variable) on each phenotypic trait (response variable). To test for potential environmental influences partitioned by sex (independent variable) in UmAsp trees, the response variables latitude, longitude, and elevation were used in separate one-way ANOVAs but sex had no significant effect on the responses (P >0.5 in all cases), therefore environmental factors were not considered necessary in analyses of phenotypic traits. In each of UmAsp and SwAsp, arthropod community composition was compared between male and female trees using non-parametric multivariate analysis of variance (npMANOVA; [63]). A Bray-Curtis dissimilarity matrix constructed from counts of arthropod herbivores on aspen genotypes (response variable) and tested for effects of tree sex (independent variable) using npMANOVA in the adonis function implemented in the R package vegan [61]. The p-value for significance was determined from 999 permutations of the data matrix.

### Gene expression analysis

#### Sample collection for microarray and RNA-Seq analysis

Sample collection from the UmAsp trees is described above and sample details are given in Additional file 5. Briefly, ten mature leaves produced from pre-formed, overwintered buds were collected per tree, from ten male and ten female trees on June 29 2009 and used to perform whole genome oligonucleotide microarray hybridisations. For RNA-Seq analysis we used a combination of a set of samples that had been collected in 2008 (five male and five female individuals collected 22-25 June) and additional samples collected in 2010 (three male and four female individuals collected 11 August). All samples consist of pools of ten leaves collected from ten buds (one leaf per bud avoiding the first and last emergent leaf) collected by removing a length of branch from either the base of the tree canopy (2009 and 2010 samples) or from a branch at a height of 4-5 m (2008 samples).

#### RNA extraction

Total RNA was extracted from 0.5 g tissue using a modified version of the CTAB method [64] as described in [65]. Briefly, the ten sampled leaves were ground under liquid nitrogen using a pestle and mortar and 0.5 g of ground material was then used for RNA extraction. Precipitated RNA was further purified using an RNeasy Mini Kit (Qiagen, Hilden, Germany) according to the manufacturer’s protocol. RNA concentration and purity were measured using a NanoDrop 2000 spectrophotometer (NanoDrop Technologies, Wilmington, DE, USA) and integrity was analysed on an Agilent 2100 Bioanalyzer (Agilent Technologies, Waldbronn, Germany). For each set of samples (*i.e.* all samples used for microarray or RNA-Seq analysis) all RNA extractions were performed together on the same day with the order of male and female samples randomised.

#### Microarray hybridisation and analysis

We used the Agilent v1.0 4x44k *Populus* gene expression oligonucleotide microarray (Agilent Technologies, Waldbronn, Germany), as detailed in the Gene Expression Omnibus platform ID GPL16040. We used the cDNA synthesis, amplification, microarray hybridisation and washing protocols supplied by Agilent (Agilent Technologies, Waldbronn, Germany) with no modifications. All hybridisations were performed using only one sample and using Cy3. Ten male and ten female individuals were profiled and the respective samples were randomised on arrays with two male and female samples run on each slide and with the position of males and females randomised between the four array sections per array slide. Arrays were scanned at 5 μm resolution, using a Scanarray 4000 microarray analysis system scanner (Perkin-Elmer, Boston, MA, USA). Spot data were extracted using GenePix (v5, Axon Instruments Inc, Union City, CA, USA). Microarray normalisation and analyses were performed using the Bioconductor [66] limma package [67] in R [62]. Microarray annotations were obtained from the PopArray resource [37] and were based on V2 of the genome annotation. The microarrays were first background corrected using the normexp method implemented in the backgroundCorrect function. Then, a between microarray quantile normalisation was performed using the normalizeBetweenArrays function. A Principle Component Analysis (PCA) plot was used for quality control and this identified one sub array assaying a female individual as a clear outlier and this sample was therefore eliminated and not used for the statistical analyses. These were conducted by fitting a linear model taking into account batch effects for slide and position of sub-array within slide to the data in order to identify genes with a high probability of differential expression between sexes. FDR-adjusted P values were used to assess the significance of differential expression.

#### RNA sequencing and analysis

Total RNA preparations were sent to the Science for Life Laboratory (SciLifeLab, Stockholm, Sweden) for sequencing. Paired-end (2 × 100 bp) RNA-Seq data were generated using standard Illumina protocols and kits (TruSeq SBS KIT-HS v3, FC-401-3001; TruSeq PE Cluster Kit v3, PE-401-3001) and all sequencing was performed using the Illumina HiSeq 2000 platform. We generated data from 8 male individuals (five sampled in 2008 and three in 2010) and 9 female individuals (five sampled in 2008 and four in 2010). For sequencing, samples were recoded (from 1-17) with males and females randomised to avoid bias due to sample handling order. Samples were multiplexed by the addition of a unique barcode sequence and all samples were profiled on two lanes of the same flowcell with male and female samples and samples from 2008 and 2010 randomised between the two lanes. Briefly, the sequencing protocol involved DNase 1 digestion of total RNA, mRNA isolation by use of oligo(dT) beads, mRNA fragmentation, first and second strand cDNA synthesis, end-repair, A-tailing, bar-coded adapter ligation and PCR amplification. Sequencing libraries were quality checked using an Agilent 2100 Bioanalyzer (Agilent Technologies, Waldbronn, Germany) before sequencing. The quality of the raw sequence data was assessed using FastQC (http://www.bioinformatics.babraham.ac.uk/projects/fastqc/). Data were then filtered to remove adapters and trimmed for quality using Trimmomatic (v0.32; [68]; settings TruSeq3-PE-2.fa:2:30:10 LEADING:3 SLIDINGWINDOW:5:20 MINLEN:50). Residual ribosomal RNA (rRNA) contamination was assessed and filtered using SortMeRNA (v1.9; [69]; settings -n 6 -a 8 -v) using the rRNA sequences provided with SortMeRNA (rfam-5 s-database-id98.fasta, rfam-5.8 s-database-id98.fasta, silva-bac-16 s-database-id85.fasta, silva-euk-18 s-database-id95.fasta, silva-bac-23 s-database-id98.fasta and silva-euk-28 s-database-id98.fasta). After both filtering steps, FastQC was run again to ensure that no technical artefacts were introduced. Filtered reads were aligned to v3.0 of the *P. trichocarpa* genome (retrieved from the Phytozome [70] resource) using STAR (v2.3.1e [71]; non default settings: --OutQSconversion -31 --outReadsUnmapped Fastx --alignIntronMax 11000). The annotations obtained from the *P. trichocarpa* v3.0 GFF file were modified to generate ‘synthetic’ gene models; *i.e.* for each gene a non-redundant set of all exons from all transcripts was defined, with overlapping exons merged where necessary. This gene-model GFF file and the OSA read alignments were used as input to the HTSeq (http://www-huber.embl.de/users/anders/HTSeq/doc/overview.html) htseq-count python utility to calculate exon-based read count values. The htseq-count utility takes only uniquely mapping reads into account. Statistical analysis of single-gene differential expression between sexes was performed in R (v3.1.0 [62]) using the Bioconductor (v2.14 [66]) DESeq and DESeq2 packages (v1.16.0 [72] and v1.4.5 [73]). For the DESeq/DESeq2 analyses, a two-factor linear model was fitted with the factors Sex and Year where Year was included as a blocking factor and the effect of Sex was tested after removal of the Year effect. FDR adjusted p-values were used to assess significance. The normalised read counts obtained from DESeq2 were used for all subsequent expression analyses, *e.g.* PCA, which were performed in R, with the exception of the differential gene expression analyses, which were performed using DESeq as it has been shown to be the most conservative of the currently available methods with the lowest false discovery rate [74]. An overview of the data, including raw and post-QC read counts and alignment rates is given in Additional file 6.

We analysed the RNA-Seq dataset using read alignments to both v2.0 and v3.0 of the *P. trichocarpa* genome assembly and annotation, yielding similar results in both cases. Similarly we analysed the microarray dataset using probe annotations based on v1.0 and v2.0 of the genome and assembly with similar gene-level results in both cases. We have also analysed the microarray data at the probe level, again yielding similar results.

#### Support vector machine identification of sex-predictive gene combinations

We used both the microarray data and normalised RNA-Seq expression values to test for the presence of contiguous gene combinations (*i.e.* windows of genes located next to each other within the genome) that were predictive of sex. We applied a sliding window across the genome with a window size of 10 genes (other window sizes were also tested with similar results). In total our expression data included 30,709 and 20,557 genes in the RNA-Seq and microarray datasets, respectively. The criterion for accepting a gene inside a window was that it had at least 5 samples with non-zero expression values. Furthermore, only windows with at least 4 accepted genes were included. The Python module scikit-learn [75] was used to train SVMs with a radial basis function (RBF) kernel parameterised by *C* and γ. This approach has previously been shown effective on gene expression data [76]. Since the optimal values of these parameters are not known prior to training, a grid search was performed in a parameter space consisting of *γ* = {10^− 4^, 5 ⋅ 10^− 4^, 10^− 3^, 5 ⋅ 10^− 3^, 10^− 2^, 10^− 1^, 1} *and C* = {1, 10, 10^3^, 5 ⋅ 10^3^, 10^4^, 5 ⋅ 10^4^, 10^5^}. For each genomic window, a double cross validation (CV) was performed where the outer CV was a leave-one-out and the inner was a 2-fold CV. The inner CV was used to train the SVM (*i.e.* estimate the parameters), and parameters with the smallest prediction error were used to predict the test data from the outer CV. The error rate was measured as the fraction of incorrect sex predictions. To validate the error rates, a permutation test was performed where 10,000 random genomic windows from all scaffolds were used in the same machine learning approach, but where the sex assignments were shuffled.

### Availability of supporting information

Microarray data has been deposited to the Gene Expression Omnibus (GEO) under the accession ID GSE46219. Raw RNA-Seq data has been deposited to the European Nucleotide Archive (ENA) under the accession ID ERP002471.

Raw RNA-Seq fastq, the synthetic exon GFF3 file used for read alignment and HTSeq analysis, read alignment BAM files and other associated outputs from the gene expression analysis can be downloaded from the PopGenIE (*Populus* Genome Integrative Explorer; [58]) FTP resource [38]). The FTP site includes RData files for both gene expression datasets as well as an HTML transcript of the analyses performed, which we highly encourage readers to examine as all analysis details are included in addition to a number of summary plots exploring the dataset. To facilitate future meta-analyses, all phenotype data used in this study is also available at the FTP site. The data pre-processing source code is available through our public git repository accessible at https://bioinformatics.upsc.se. The RNA-Seq expression data presented here has been integrated in the exImage and exPlot expression visualisation tools at PopGenIE.org [58], where they are called the “Expression diversity (RNASeq)” dataset.

## Additional files

Additional file 1
**Statistical analyses of phenotypic and biochemical traits in the UmAsp and SwAsp samples.** Phenotypic trait means and standard deviations for female and male individuals from the UmAsp collection (sheet1) and the SwAsp collection (sheet2), with results of one-way ANalyses Of VAriance (ANOVA). Where data could not be transformed to meet the assumptions of variance structure for ANOVA, a Mann–Whitney *U* test was conducted. For the extended phenotype of the arthropod community, non-parametric MANOVA (npMANOVA) results are shown for each of the UmAsp collection (sheet 1) and SwAsp collection (sheet 2). Additional file 2
**PDF image containing dot plot representations of per-sample expression values of the four genes with the smallest p values (regardless of significance) when testing for the effect of sex (top row), the four genes with the highest fold-change between males and females (middle row) and the lowest fold change (bottom row).** Bold text gene identifiers in the top row of plots indicate the two statistically significant genes. The genes represented in these figures are those circled in red in Figure 4b. Expression values represent variance stabilising transformation normalised read counts derived using HTSeq and DESeq2. Black lines represent the median expression value per sex. For each gene male and female samples are plotted separately with males represented by blue dots and females by pink dots. The position along the x-axis of the plot has no meaning and merely separates male from female samples. Note that the y-axis is a log scale, for which a pseudo count was added to every value to avoid infinite values from the log transformation. Additional file 3
**PDF file containing individual plots of per base pair read coverage for reads aligning uniquely to the Potri.019G047300 locus.**
Additional file 4
**PDF file containing plots further exploring sex ratio of individuals and populations in the Swedish Aspen collection in relation to elevation, latitude and marker based population structure.**
Additional file 5
**Sex, longitude and latitude of clone origin for UmAsp and SwAsp, and elevation for UmAsp, samples used in the current study.** The year of sampling for phenotype, microarray and RNA-Seq analysis is indicated. For UmAsp trees the longitude, latitude and elevation values represent the location of the actual tree sampled. For SwAsp samples they represent the origin of the original clone that was used to establish the clonal common garden experiment at the Skogforsk research station, Sävar, near Umeå, (63.896054°N, 20.549321°E). All UmAsp clones flowered in 2007. For the SwAsp samples, the number of clonal replicates present in the common garden is shown. Additional file 6
**Overview of RNA-Seq data including quality control metrics and correspondence between sample and ENA submission IDs.**

## Abbreviations

cDNA: Complementary DNA
CDS: Coding DNA sequence
DNA: Deoxyribonucleic acid
ENA: European nucleotide archive
FTP: File transfer protocol
GEO: Gene expression omnibus
GFF: General feature format
GO: Gene ontology
PCA: Principal component analysis
QA: Quality assessment
rRNA: Ribosomal RNA
RNA: Seq – RNA-sequencing
RNA: Ribonucleic acid
SVM: Support vector machines
SwAsp: Swedish aspen
UmAsp: Umeå aspen
VST: Variance stabilising transformation
