# Supplementary figures and images for: Populus tremula (European aspen) shows no evidence of sexual dimorphism
Source: BMC Plant Biol. 2014 Oct 16;14:276. doi: 10.1186/s12870-014-0276-5 (PMC4203875; doi:10.1186/s12870-014-0276-5)

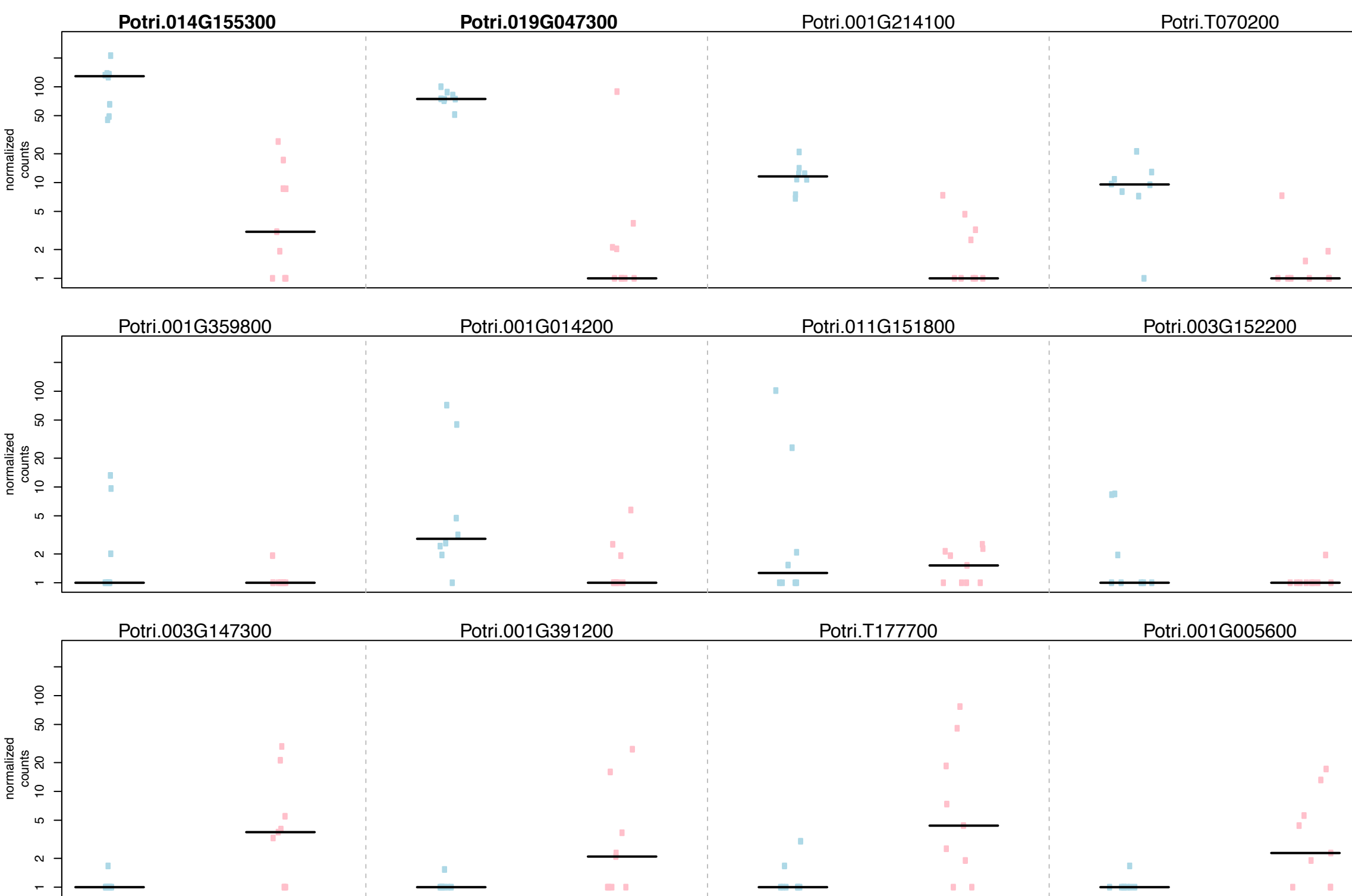

Supplement: Additional file 2 — PDF image containing dot plot representations of per-sample expression values of the four genes with the smallest p values (regardless of significance) when testing for the effect of sex (top row), the four genes with the highest fold-change between males and females (middle row) and the lowest fold change (bottom row). Bold text gene identifiers in the top row of plots indicate the two statistically significant genes. The genes represented in these figures are those circled in red in Figure 4b. Expression values represent variance stabilising transformation normalised read counts derived using HTSeq and DESeq2. Black lines represent the median expression value per sex. For each gene male and female samples are plotted separately with males represented by blue dots and females by pink dots. The position along the x-axis of the plot has no meaning and merely separates male from female samples. Note that the y-axis is a log scale, for which a pseudo count was added to every value to avoid infinite values from the log transformation. [file 12870_2014_276_MOESM2_ESM.pdf]

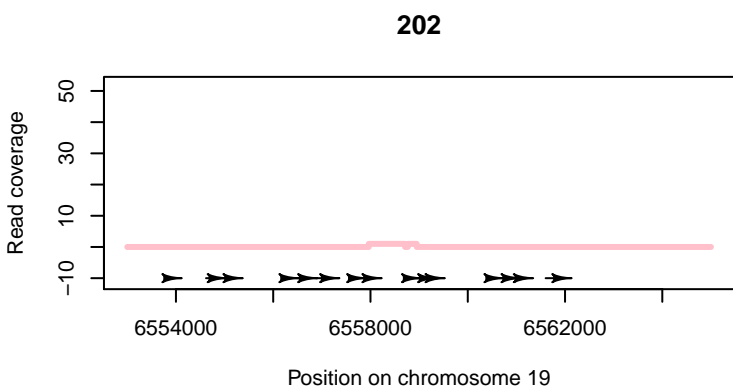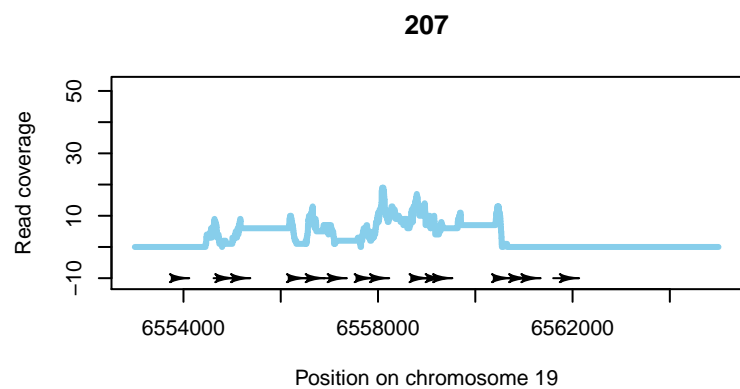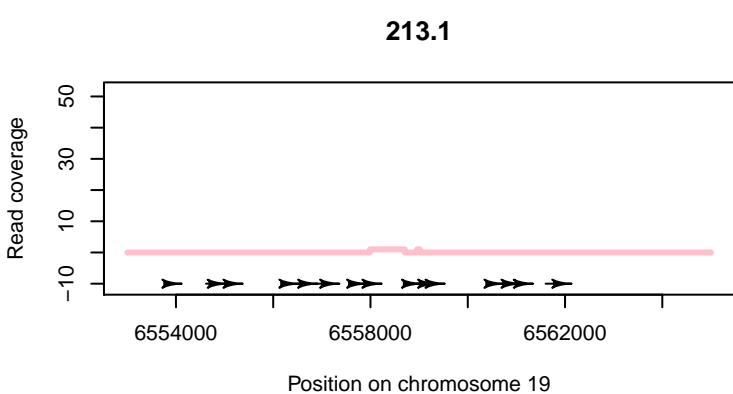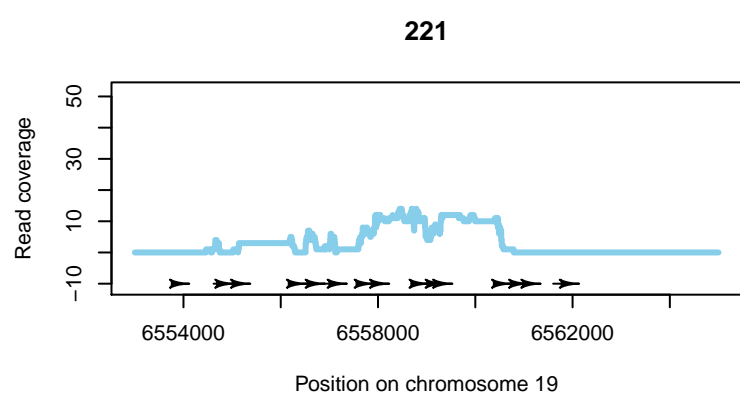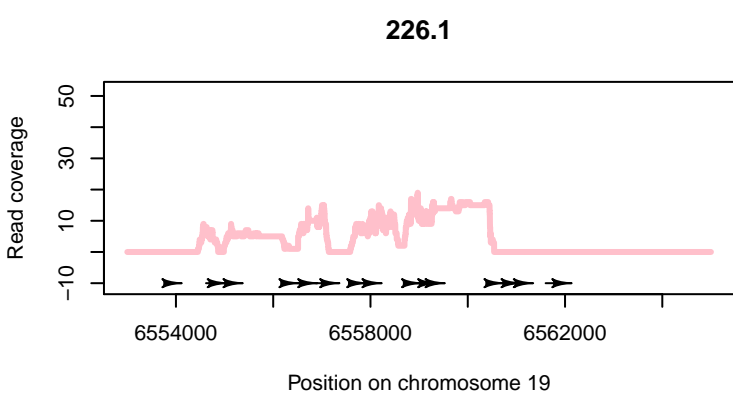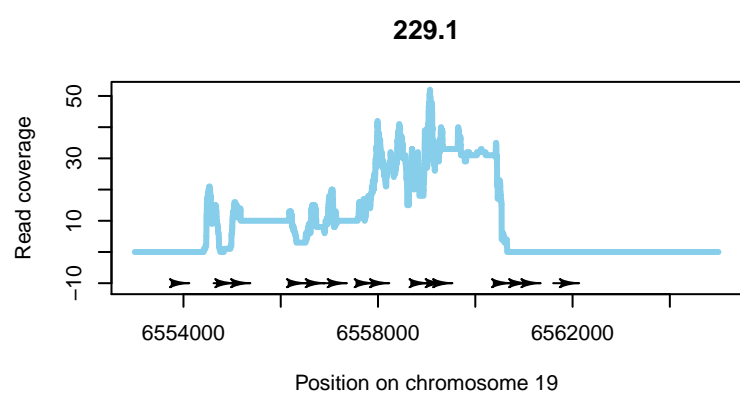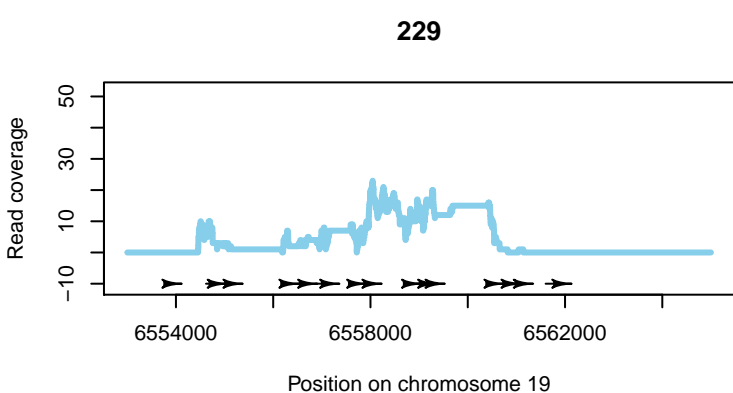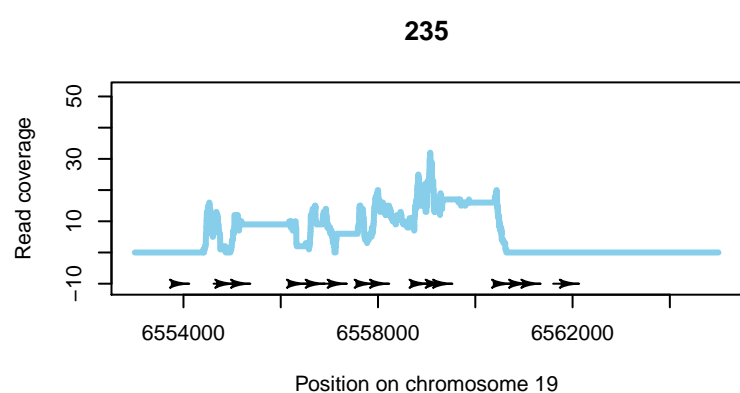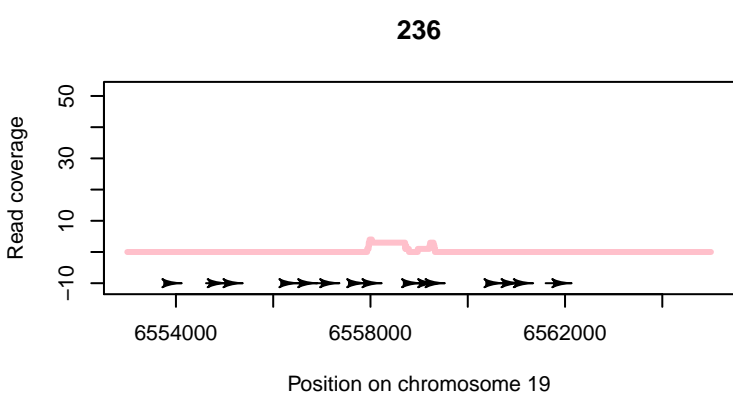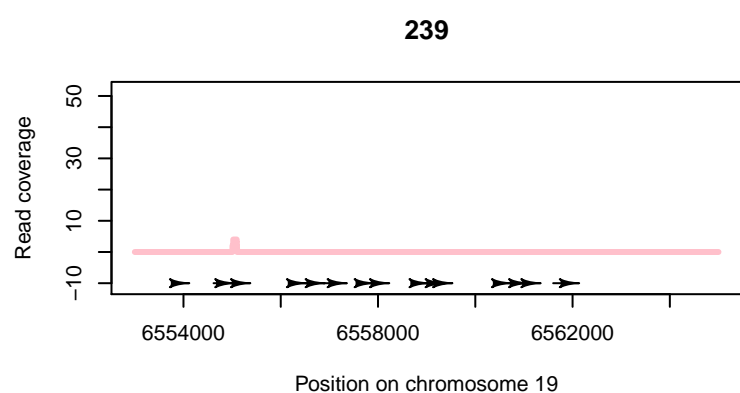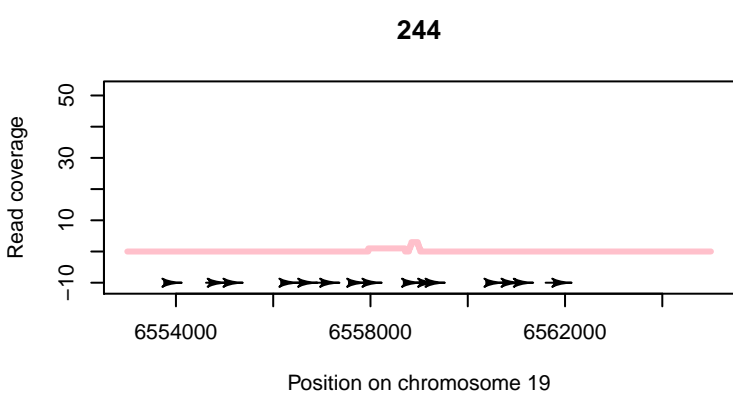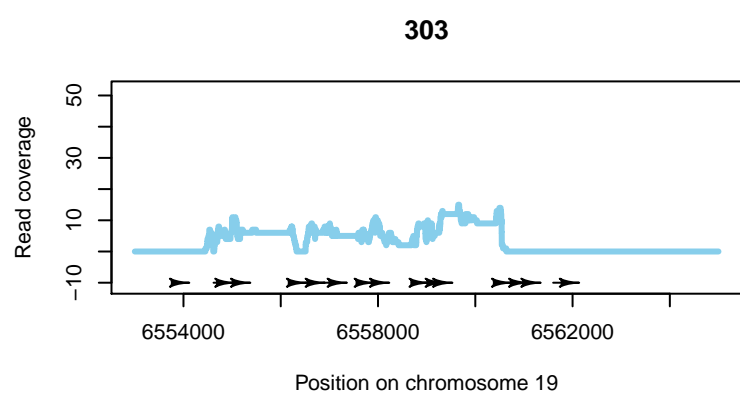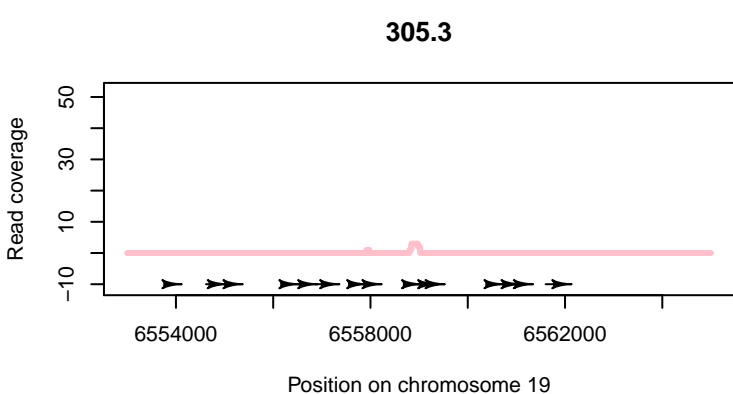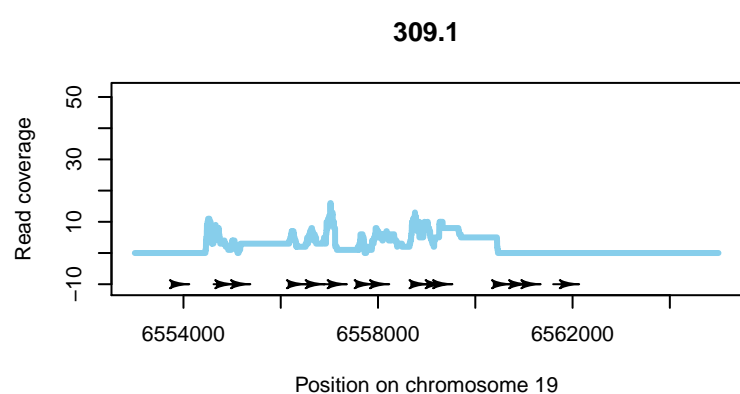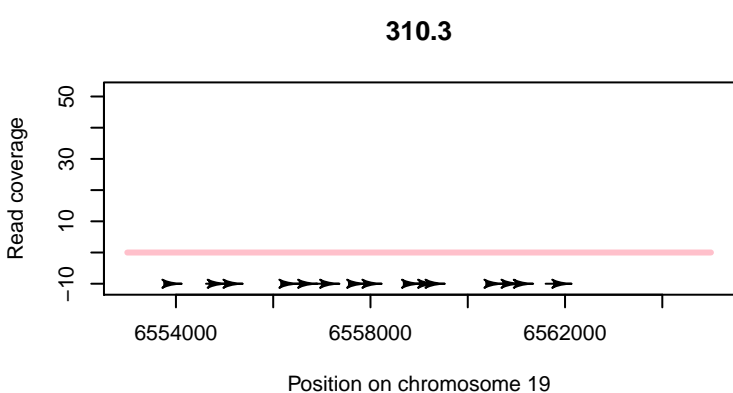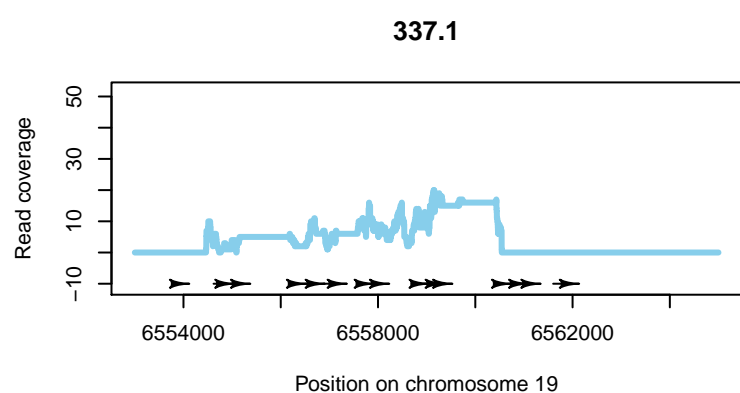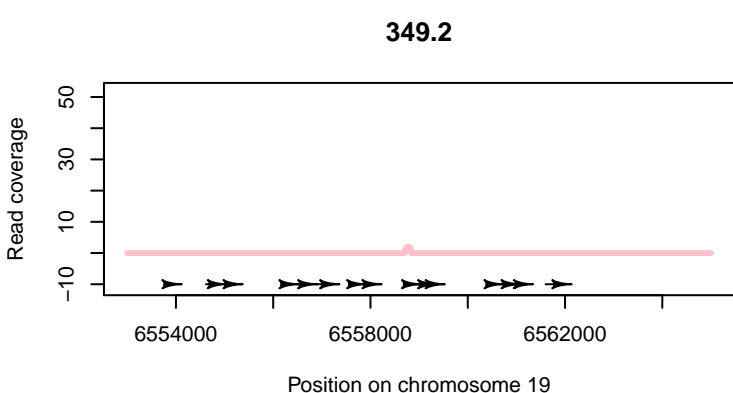

Supplement: Additional file 3 — PDF file containing individual plots of per base pair read coverage for reads aligning uniquely to the Potri.019G047300 locus. [file 12870_2014_276_MOESM3_ESM.pdf]
