# Supplementary material for: Populus tremula (European aspen) shows no evidence of sexual dimorphism
Source: BMC Plant Biol. 2014 Oct 16;14:276. doi: 10.1186/s12870-014-0276-5 (PMC4203875; doi:10.1186/s12870-014-0276-5)
Supplement: Additional file 4 — PDF file containing plots further exploring sex ratio of individuals and populations in the Swedish Aspen collection in relation to elevation, latitude and marker based population structure. [file 12870_2014_276_MOESM4_ESM.pdf]

Sex ratios in SwAsp

All genotypes of known sex growing at < 50 m (n=14) or > 300 m (n=4) elevation were extracted and plotted as Low elevation or High elevation.

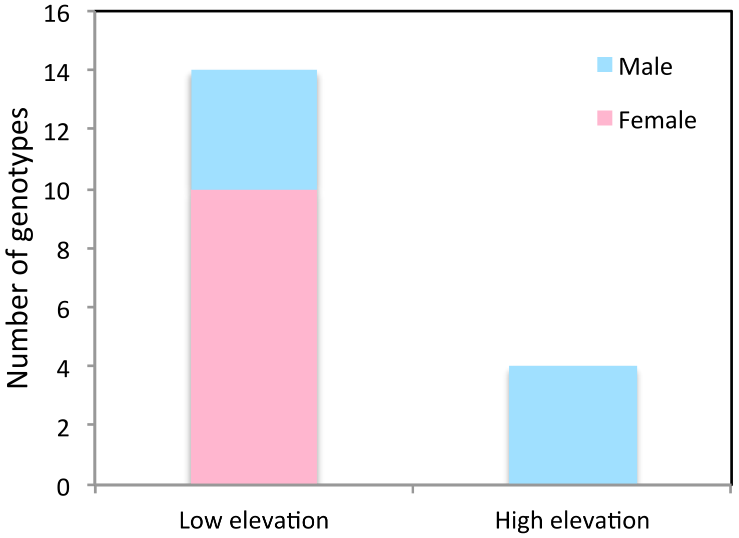

Populations are plotted from north to south on the X-axis (population latitudes). The number of female, male and unknown sex genotypes is stacked in each bar for each population.

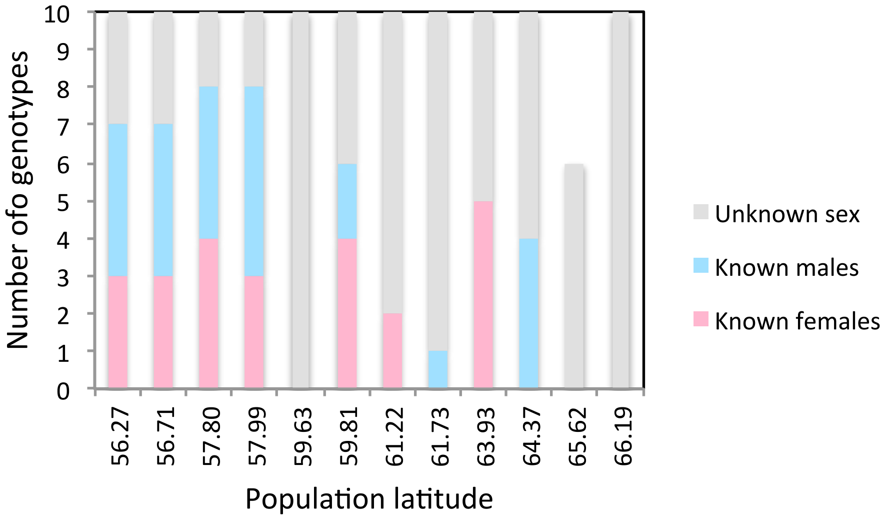

Clusters of genotypes from south, central and north regions of Sweden were identified by Bernhardsson et al. [1] based on SNPs. The proportion of known females and males in each of the genotype groups are plotted as stacked bars.

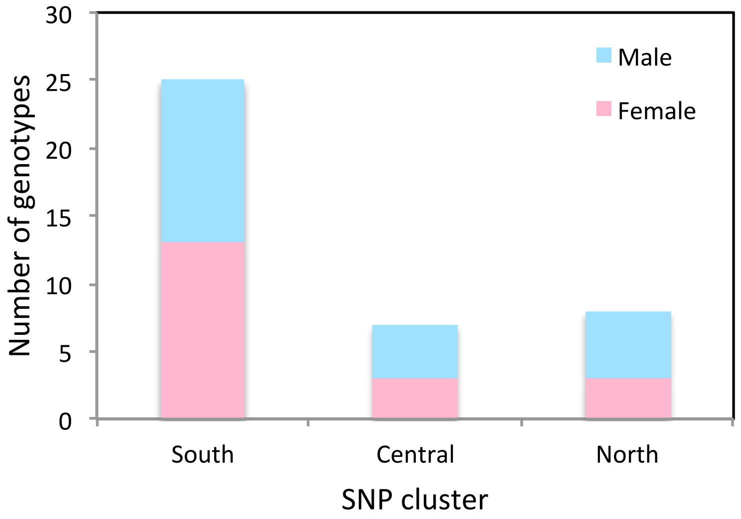

Wilcoxon tests for differences in tree longitude and latitude of origin, where tree sex was a factor, were each non-significant. A Wilcoxon test to test for differences for elevation of tree origin where tree sex was a factor was significant (P = 0.044).

1. Bernhardsson C, Robinson KM, Abreu IN, Jansson S, Albrectsen BR, Ingvarsson PK: **Geographic structure in metabolome and herbivore community co-occurs with genetic structure in plant defence genes.** *Ecol Lett* 2013, **16**:791–8.
